# Synthetic Mucins as Glycan-Defined Prebiotics

**DOI:** 10.1021/acscentsci.5c00317

**Published:** 2025-06-02

**Authors:** Jill W. Alty, Carolyn E. Barnes, Agnese M. Nicoli, Bradley S. Turner, Ekua A. Beneman, Amanda E. Dugan, Spencer D. Brucks, Austin G. Kruger, Richard R. Schrock, Katharina Ribbeck, Laura L. Kiessling

**Affiliations:** † Department of Chemistry, 2167 Massachusetts Institute of Technology, Cambridge, Massachusetts 02139, United States; ‡ Department of Biological Engineering, 2167 Massachusetts Institute of Technology, Cambridge, Massachusetts 02139, United States; § Department of Chemistry, University of California at Riverside, Riverside, California 92507, United States; ∥ The Koch Institute of MIT, Cambridge, Massachusetts 02142, United States; ⊥ The Broad Institute of MIT and Harvard, Cambridge, Massachusetts 02142, United States

## Abstract

The human microbiome contains at least as many bacterial cells as human cells. Some bacteria offer benefits, like improving gut barrier function, suppressing pathobiont growth, and modulating immunity. These benefits have popularized probiotics, but probiotic retention is often hindered by low colonization efficiency in the mucosal layer that lines all epithelial cells. Mucins, the primary components of mucus, are essential for the organization and regulation of microbial populations. The molecular mechanisms of mucin–probiotic interactions remain understudied due, in part, to the inability to incisively manipulate native mucin sequences or their glycans. Here, we used synthetic mucins with defined glycan presentations to interrogate glycan-dependent interactions between mucus and three probiotic lactobacilli species. The nutrient conditions under which bacteria were cultured influenced glycan binding preferences, suggesting mucin–probiotic interactions change with nutrient availability. The addition of synthetic mucins to native mucin increased *Limosilactobacillus fermentum* adherence. Additionally, an increase in glycosidase activity indicated that native and synthetic mucins function as prebiotics, as probiotic bacteria can cleave the displayed *O*-glycans. Thus, synthetic mucins can cultivate target probiotic bacteria and increase adhesion as binding sites, highlighting their value as tools for elucidating native mucin functions and as promising agents for promoting human health.

## Graphical abstract

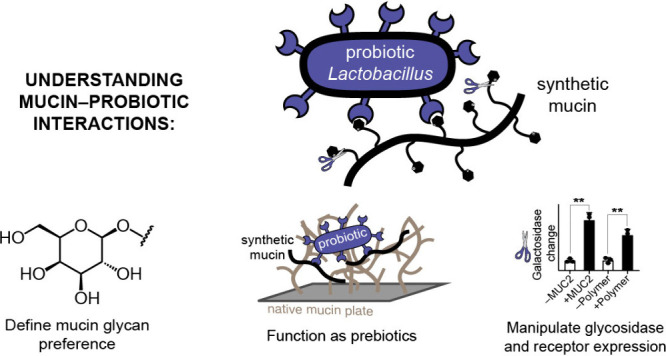

## Introduction

Trillions of bacteria are housed within the outer mucus layer in the gut, encompassing disease-causing pathogens or beneficial commensals.^1–3^ Some bacteria are probiotic, defined as live microorganisms that offer a health benefit to the host.^4^ Regarded as valuable probiotics, lactobacilli are often found in exogenous sources like fermented food products.^5–7^ Studies indicate that lactobacilli mitigate antibiotic-associated diarrhea, alter the pathogenicity of other microbes, and regulate host immunity by reducing inflammation and activating immune cells.^8–12^ Lactobacilli can produce short-chain fatty acids through the fermentation of carbohydrates, improving gut microbiota dysbiosis, promoting phage production, and, in some cases, eliciting anti-tumor responses.^13–16^ Supplementation with probiotic lactobacilli is therefore a promising strategy for maintaining a healthy gut microbiome and treating conditions like irritable bowel disease or bacterial infection.^17–20^ However, the retention of probiotic bacteria is challenging as they are typically cleared from the body in three to 10 days due to their low mucosal adhesion and colonization efficiency.^21,22^

Mucins, the main structural components of the mucosal layer, are a family of proteins characterized by their extended backbone conformation and dense *O*-glycosylation.^23,24^ The structural characteristics of mucins and their *O*-glycosylation patterns are essential for organizing, cultivating, and regulating microbial populations by adhering microbes and serving as nutrient sources (Figure 1A).^25^ These functions support the growth of mucus-binding probiotics, such as lactobacilli.^26–33^

**Figure 1:**
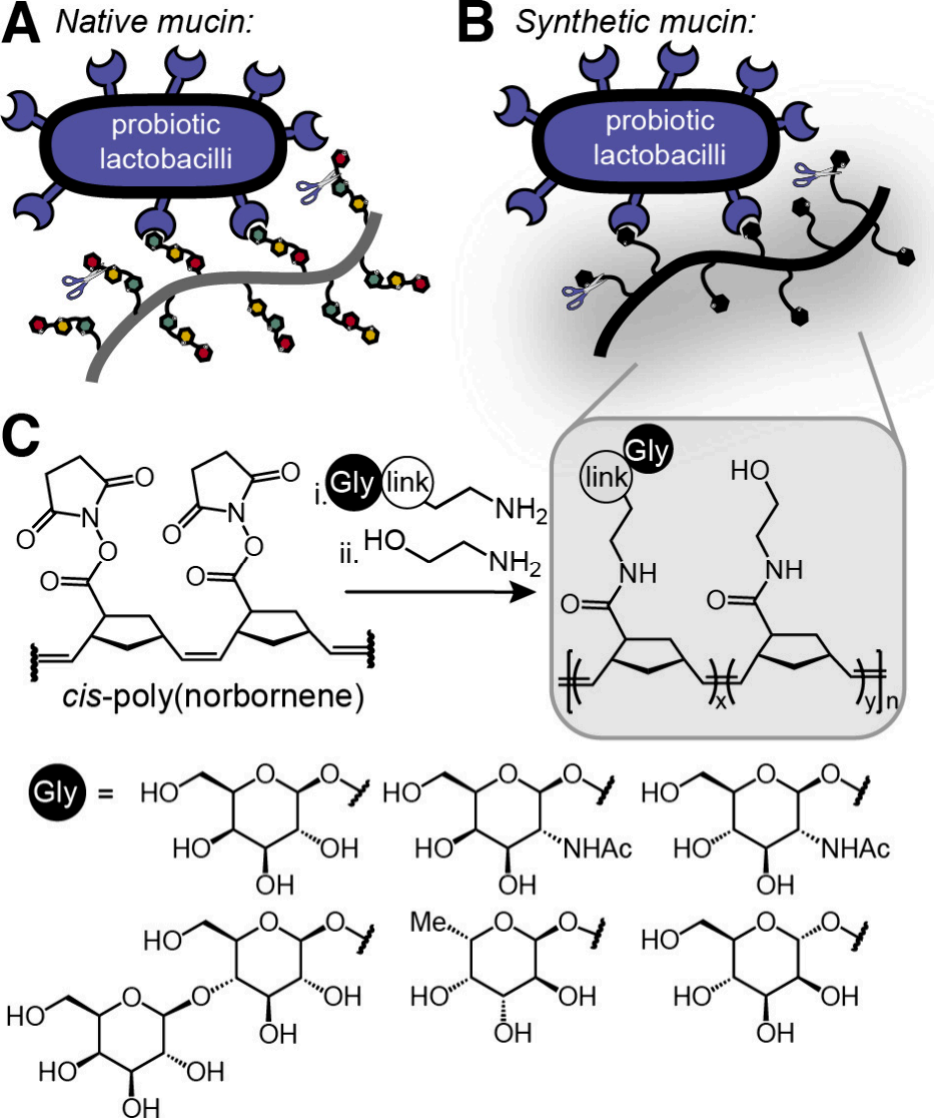
A) Native mucins as prebiotics for adhesion and nutrition of probiotics. B) Synthetic mucins as prebiotics. C) Mucin surrogate synthesis. Link is ethanolamine, triethylene glycol, or aryl (see Supporting Information for details).

As key nutrient sources and adhesion sites for gut bacteria, mucins may be regarded as prebiotics, defined as “a substrate that is selectively utilized by host microorganisms conferring a health benefit”.^4,34–36^ Prebiotics serve as nutrients for probiotics, encouraging bacterial production of short-chain fatty acids and modulating immune function through their interactions with toll-like receptors and commensal bacteria.^4,37^ Common prebiotics include galacto- and fructo-oligosaccharides, though mucin glycans have also been shown to be effective prebiotics.^38^ The use of mucins as prebiotics has emerged as a strategy to manipulate the gut microbiome. This approach may more effectively diversify microbial community composition than live probiotic supplementation.^36^

A critical feature of mucins is their multivalent display of glycans. While it is understood that bacteria bind native mucins via their *O*-glycans, the inability to manipulate the chemical structure of native mucins complicates efforts to identify the features responsible. Despite advances, challenges remain, including difficulties in recombinantly expressing native mucins, the inability to genetically control mucin glycosylation, and the complexity of synthesizing mucin glycans.^39–42^ Synthetic polymers displaying defined glycans can function as mucin mimetics, overcoming the challenges of unknown and heterogeneous glycosylation patterns.^43–46^ To date, studies with synthetic mucins have focused on inhibiting pathogenic bacteria or virulence pathways. We envisioned that synthetic mucins could act as prebiotics to enhance probiotic adhesion and inform on the binding preferences of probiotics (Figure 1B).

Herein, we synthesize a suite of chemically defined synthetic mucins to probe their function as prebiotics for probiotic lactobacilli. These mimics display terminal saccharides of native mucin on an extended synthetic backbone (Figure 1C). We generated compounds that bound a group of probiotics that included *Lactiplantibacillus plantarum*, *Limosilactobacillus fermentum*, and *Limosilactobacillus reuteri*, all of which are found in probiotic formulations. Our data reveal the glycan-binding preferences for each organism. Bacterial binding to synthetic mucins was highly dependent on the nutrient sources available during growth, with nutrient-rich media leading to glycan-specific binding. In contrast, nutrient-poor media resulted in more promiscuous binding, highlighting how the environment influences the ability of probiotics to adhere to these mucin-like compounds. The multivalent display of the mimetics induced clustering and adhesion of the bacterial species. When *L. fermentum* was incubated in the presence of both native and synthetic mucins, more probiotic bacteria adhered to the mucosa, indicating that synthetic mucins were prebiotic. We also detected glycosidase production, which leads to *O*-glycan cleavage, presumably further cultivating probiotic growth. This report reveals that mucin surrogates can have dual roles. First, they provide insight into the features of native mucins that facilitate their binding to probiotic bacteria. Second, mucin mimics can function as prebiotics.

## Results

### Design of Synthetic Mucins

To identify the monosaccharides involved in the interactions between mucins and lactobacilli, a collection of amine-terminated, *O*-glycan epitopes was synthesized (Figure 1C). These epitopes represented the most prevalent neutral glycan building blocks within native mucin glycan sequences: α-fucose (α-Fuc), β-galactose (β-Gal), β-*N*-acetyl galactosamine (β-GalNAc), β-*N*-acetyl glucosamine (β-GlcNAc), as well as the disaccharide lactose (β-Lac).^30,47^ Though present in trace quantities on native mucins in *N*-linked glycans,^48^ we included α-mannose (α-Man) residues in this survey as the epitope has been identified to bind *L. plantarum* and *L. fermentum*.^49–54^ We typically linked the saccharide residues to the polymer scaffold through a triethylene glycol (PEG_3_) group to minimize steric effects and thereby facilitate bacterial binding.^55^

The polymeric backbone used for all synthetic mucins studied was synthesized using a *cis*-selective ring-opening metathesis polymerization of a norbornene derivative to provide an extended scaffold that mimics the morphology of native mucins, increases the steric accessibility of the glycans, and improves water solubility.^43^ Each glycan was installed by exposing a glycan building block with an amine-terminated anomeric linker to the polymer bearing pendent *N*-hydroxy succinimidyl esters (Figure 1C).^56^ The degree of glycan substitution was varied (20–80%), and as anticipated, lower glycan densities tend to result in optimal binding of the bacteria (Figure S1).^43,57,58^ For visualizing the mimetics, Alexa Fluor 405 (AF405) cadaverine was added prior to quenching any remaining succinimidyl esters with ethanolamine, such that approximately one fluorophore per chain was installed (Figure 2A). We focused on 100-mer synthetic mucins decorated with ∼20–50% glycan (Figure S2).

**Figure 2:**
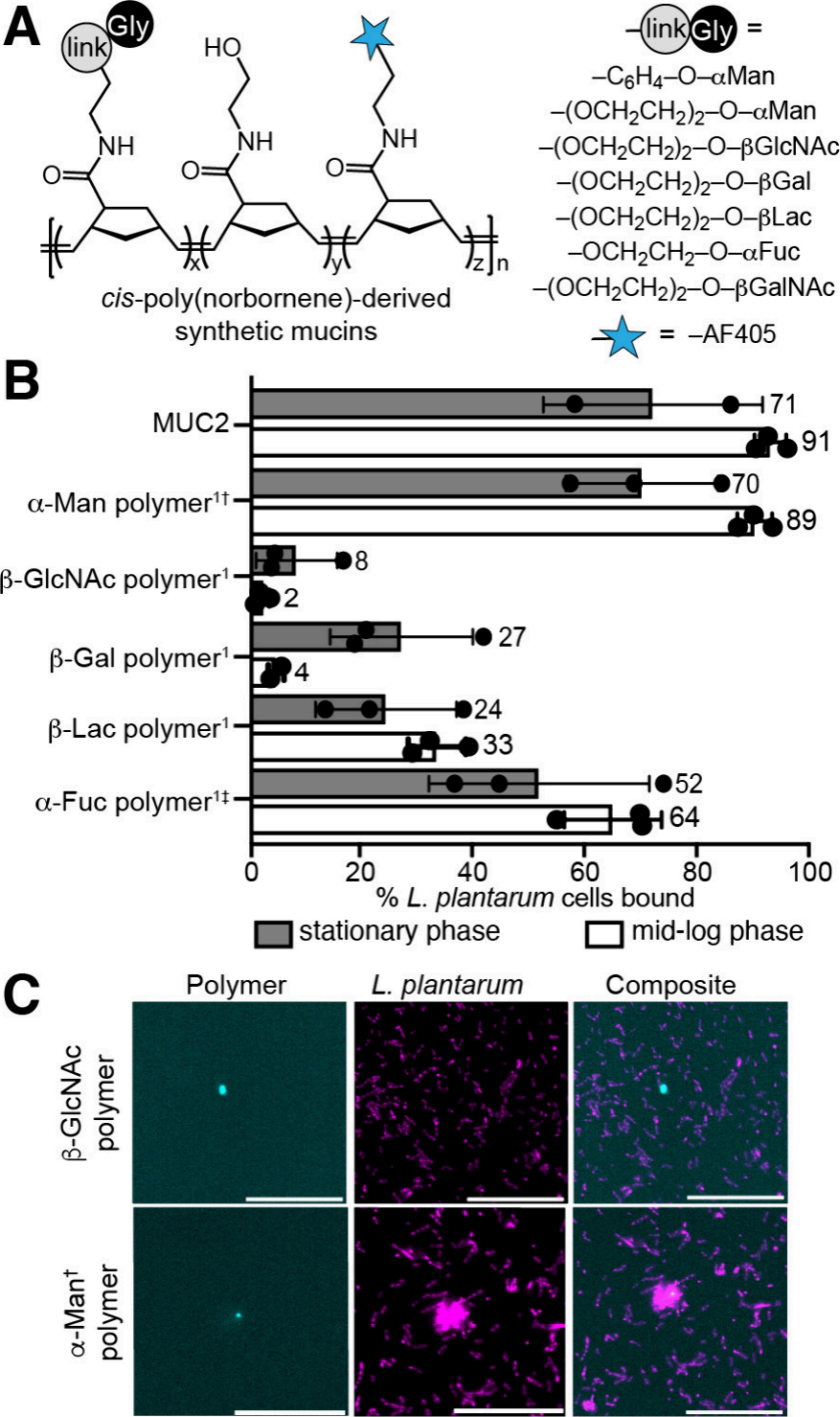
A) Synthetic mucins used in the study. Polymer molecular weights were 20–60 kDa prior to glycan attachment. Polymers were synthesized on a 10–20 mg scale. B) *L. plantarum* was purchased from ATCC. The percentage of cells bound calculated from flow cytometry of native and synthetic mucins with *L. plantarum* grown to stationary or mid-log phase in MRS broth aerobically at 37 °C. Concentrations: ^1^2 mM glycan for polymers; 0.04–0.1 wt % for MUC2. Saccharide linker is PEG_3_ unless † for aryl or ‡ for ethanolamine. Functionalizations: β-Gal = 40%, β-Lac = 20%, β-GlcNAc = 40–50%, α-Fuc = 30%, α-Man = 20%. C) Confocal microscopy of synthetic mucin and bacteria. Magenta: bacteria in stationary phase; Cyan: polymer. Scale bar, 40 μm. Data are representative of biological replicates (n ≥ 3).

### Glycan-Specific Binding of Synthetic Mucins to *L. plantarum*

To test whether bacteria interact with the polymers, we first examined *Lactiplantibacillus plantarum* NCIMB 8826. Because *L. plantarum* binds native mucus and α-mannose through mucus-binding proteins and mannose-specific adhesins, we used this probiotic to test the utility of a flow cytometry-based assay to assess mucin-mimetic binding to bacteria.^49,50,59–62^ Native or synthetic mucins were incubated with live bacteria, and then the cells were washed to remove unbound glycopolymer. The percentage of cells bound to the AF405-containing polymer was then determined by cellular fluorescence.

Mannosylated synthetic mucins served as positive control compounds and exhibited dose-dependent binding to *L. plantarum* (Figure S4). Polymers substituted with an aryl-mannoside (α-ArMan) were compared to those with an α-mannoside (α-PEG_3_Man) appended through an anomeric substituent to probe the linker’s impact. Indeed, different affinities were observed for α-ArMan polymers versus α-PEG_3_Man polymers binding to *L. plantarum*. To ensure that ligands occupied similar binding sites to mucin *O*-glycans, we generally employed glycan substituents with oligoethylene glycol linkers. Therefore, we validated that glycan binding preferences could be assessed using flow cytometry with *L. plantarum* and mannose-functionalized mucin surrogates.

*L. plantarum* binding to the collection of synthetic mucins was compared to its interaction with native gut mucin, MUC2 (Figure 2B, Figures S5–S7). Growth phase and available nutrients may affect probiotic bacteria’s fitness and their ability to colonize the human gut.^63^ Hence, we compared the probiotic binding when grown to stationary phase, or when growth has reached saturation, versus in mid-logarithmic (mid-log) phase, when growth is exponential. In both phases, *L. plantarum* cells showed excellent binding to MUC2 with increased binding in mid-log phase. For synthetic mucins bearing a single glycan epitope, α-Man polymer and α-Fuc polymer exhibited the strongest binding, agnostic of growth phase. Visualizing by confocal microscopy, the α-Man and α-Fuc synthetic mucins displayed the most coincidence of polymer and *L. plantarum* fluorescence, whereas no colocalization was observed for those synthetic mucins that flow cytometry indicated could not bind (Figure 2C).

### Growth Conditions Impact the Binding of Synthetic Mucins to *L. reuteri*

We hypothesized that the mucin surrogates could be used to determine whether glycan-binding preferences change under different growth conditions. When grown in nutrient-rich conditions (MRS broth) to stationary or mid-log phase, *Limosilactobacillus reuteri* F275 bound specifically to synthetic mucins displaying β-Lac or α-Fuc as judged by flow cytometry (Figure 3A, Figures S8 and S9) and confocal microscopy (Figure 3C).

**Figure 3:**
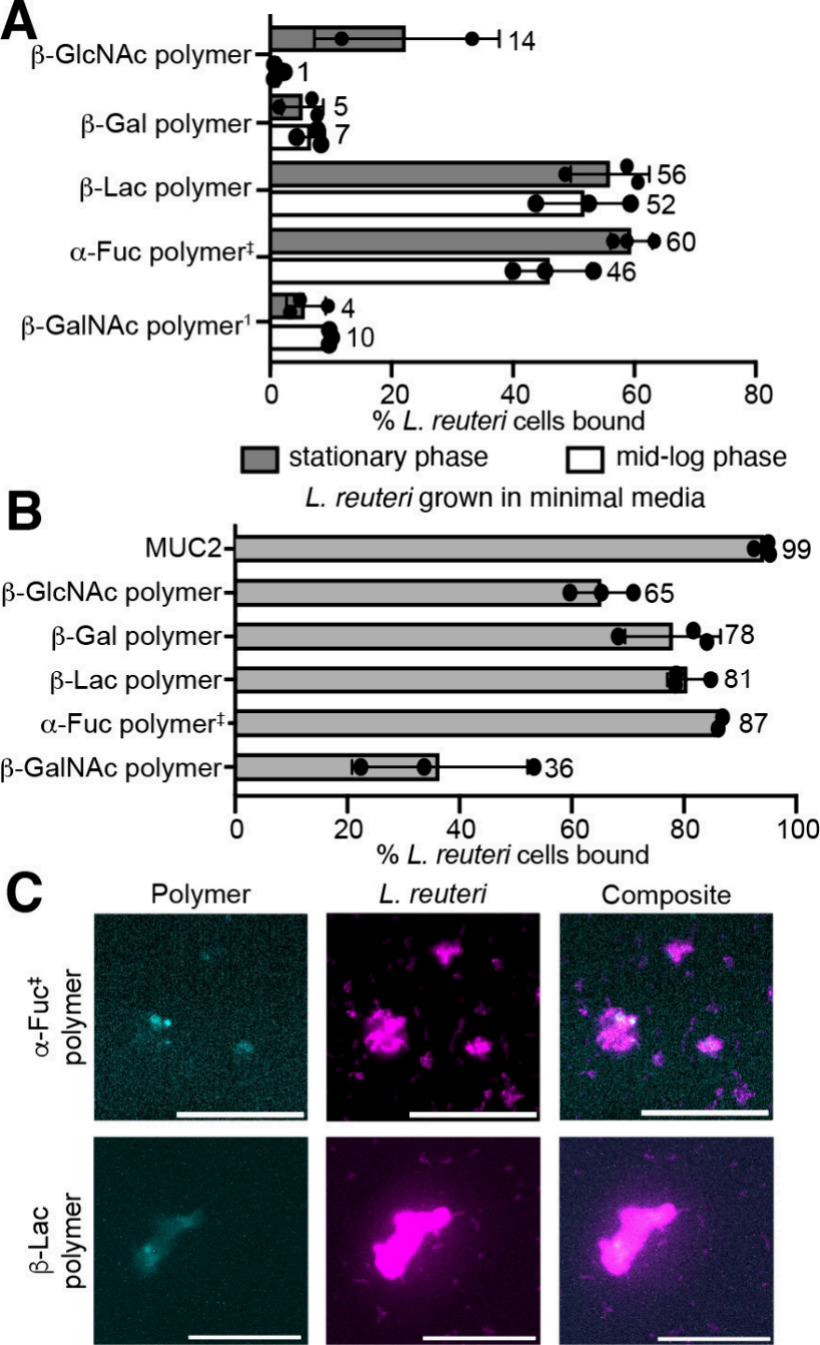
Percentage of cells bound calculated from flow cytometry of native and synthetic mucins with *L. reuteri* (from ATCC) grown in MRS broth (A) or in minimal media (B) anaerobically at 37 °C. Concentrations: 2 mM glycan for polymers, 0.02–0.06 wt % for MUC2. Saccharide linker is PEG_3_ unless ‡ for ethanolamine. Functionalizations: β-Gal = 40–45%, β-Lac = 10–20%, β-GlcNAc = 40–50%, α-Fuc = 25–50%, β-GalNAc = 30%. ^1^Concentration in stationary phase: 0.5 mM. C) Confocal microscopy of synthetic mucin and bacteria. Magenta: bacteria in stationary phase; Cyan: polymer. Scale bar, 40 μm. Data are representative of biological replicates (n ≥ 3).

Physiological settings can be nutrient-poor. We hypothesized bacteria grown in such conditions would bind a greater variety of synthetic mucins to enhance their ability to colonize a nutrient-scarce gut.^63^ Indeed, when bacteria were cultured in minimal media (Table S1), mucin binding by *L. reuteri* was enriched. Moreover, the previously observed glycan preferences were not apparent, as *L. reuteri* binding was enhanced for all mucin surrogates (Figure 3C, Figure S10).

### Influence of Growth Phase on Mucin Interactions with *L. fermentum*

Probiotic *Limosilactobacillus fermentum* Beijerinck 36 is closely related to *L. reuteri* and is known to express mucin-binding proteins.^26,64–66^ Like *L. reuteri*, *L. fermentum* bound most avidly to synthetic mucins bearing β-lactose residues. When this species was grown to mid-log phase, it was less selective, binding most synthetic mucins (Figure 4A, Figure S12). Synthetic mucins presenting β-lactose bound 97% of *L. fermentum*, outperforming native mucin. Confocal microscopy revealed that synthetic mucins bearing α-Man and β-Gal colocalize with *L. fermentum*, further supporting the glycan specificity observed with flow cytometry (Figure 4B). As bacterial growth slowed in the stationary phase, the probiotic’s affinity for synthetic mucins diminished, but native mucins retained excellent binding (Figure 4A, Figure S13).

**Figure 4:**
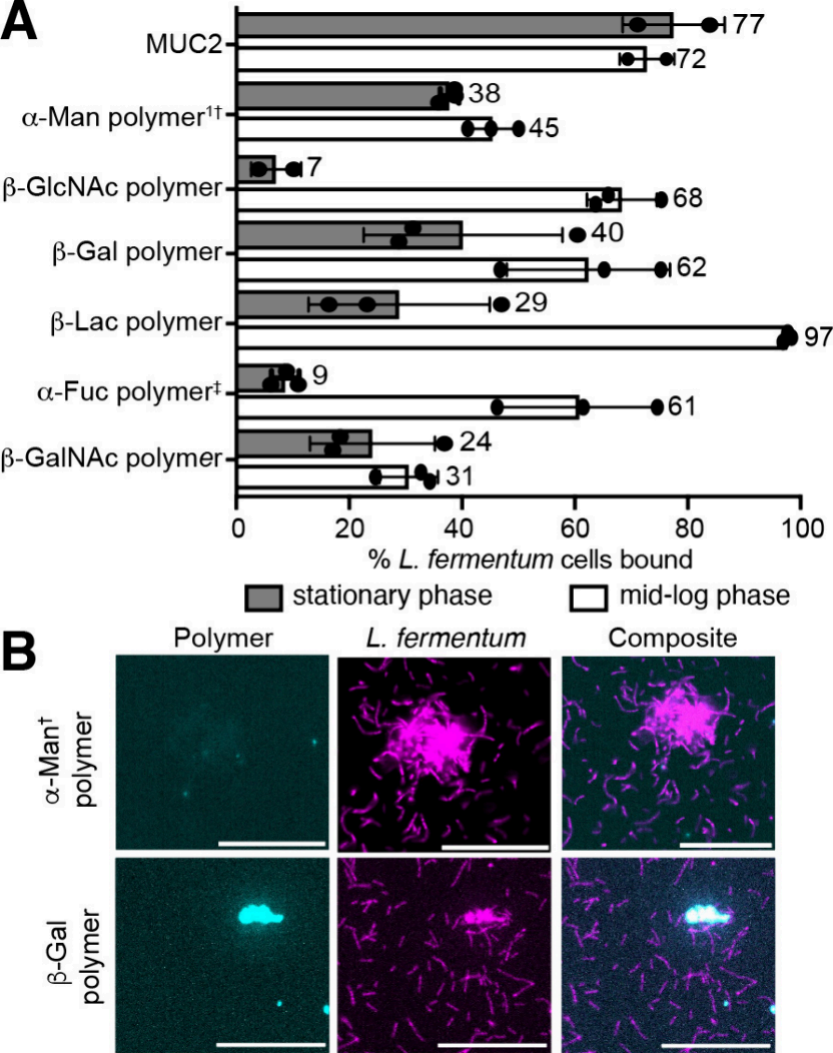
A) Percentage of cells bound calculated from flow cytometry of native or synthetic mucins with *L. fermentum* (from ATCC) cultured in MRS broth aerobically at 37 °C to stationary or mid-log phase. Concentrations: 2 mM glycan for polymers, 0.02–0.04 wt % for MUC2. Saccharide linker is PEG_3_ unless † for aryl or ‡ for ethanolamine. Functionalizations: β-Gal = 40–45%, β-Lac = 15–20%, β-GlcNAc = 40–50%, α-Fuc = 25–30%, β-GalNAc = 30%, α-Man = 20–40%. ^1^Concentration in stationary phase: 0.5 mM. B) Confocal microscopy shows colocalization of synthetic mucin and bacteria. Magenta: bacteria in stationary phase; Cyan: polymer. Scale bar, 40 mm. Data are representative of biological replicates (n ≥ 3).

### Clustering of Lactobacilli through Multivalency

Because native and synthetic mucins both present multivalent arrays of glycans, they can engage multiple bacteria to promote cell clustering or aggregation. To quantify the extent of bacterial clustering by native and synthetic mucins, we analyzed the size of cell clusters using microscopy.^67^ For each species, the synthetic mucin that bound the most bacterial cells also produced the largest average cluster size (Figures S15–S17). When *L. plantarum* was exposed to polymers bearing α-Fuc or α-Man, the population of single cells decreased by 50% or 60%, respectively, and the cell cluster size ranged up to 5 microns (Figure S15). In contrast, synthetic mucins bearing β-GlcNAc, a non-binding epitope, had no effect on microbial clustering. MUC2 modestly agglutinated *L. fermentum*, while synthetic mucins presenting β-Gal or α-Man decreased the population of single cells, giving rise to a broad dispersity of bacterial clusters (Figure 5A, Figure S17). We envisioned leveraging this outcome to enhance bacterial attachment to mucus.

**Figure 5:**
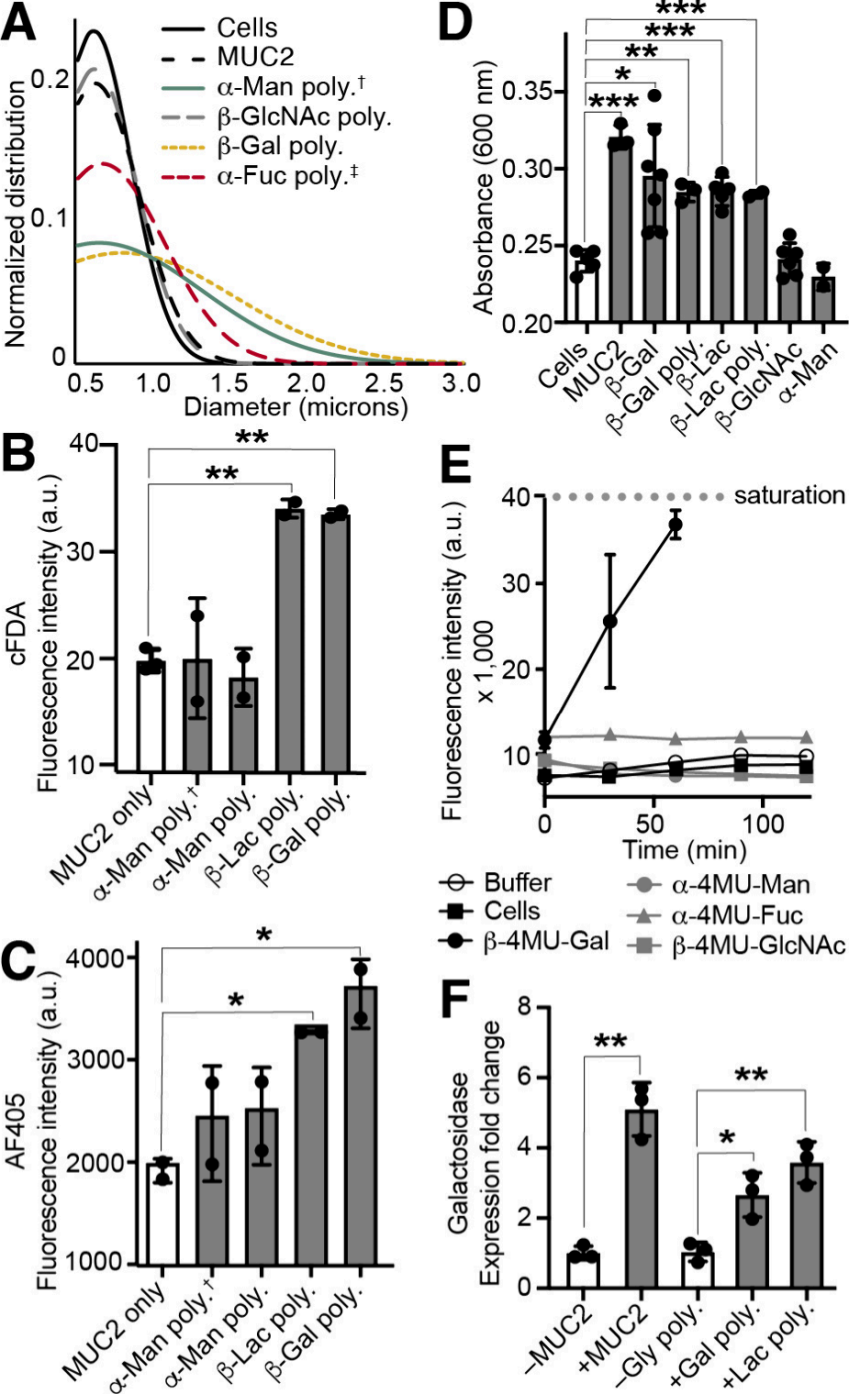
A) *L. fermentum* cellular clustering by native and synthetic mucins visualized by confocal microscopy and quantified using Fiji software. Saccharide linker is PEG_3_ unless † for aryl or ‡ for ethanolamine. B) *L. fermentum* adhesion to MUC2-coated wells in the presence or absence of synthetic mucins measured by cFDA fluorescence. C) Synthetic mucin retention to MUC2-coated wells with *L. fermentum* measured by AF405 fluorescence. D) Nutritional glycan preferences of *L. fermentum* in minimal media with mono- or disaccharides as monitored by adhesion assay. E) Glycosidase activity of *L. fermentum* as monitored by fluorogenic glycans. F) qPCR analysis of β-galactosidase expression for *L. fermentum* grown in the presence or absence of native or synthetic mucins. Statistical analysis was performed using ANOVA with multiple comparisons test: *P < 0.1, **P < 0.01, ***P < 0.001. Data are representative of biological replicates (n ≥ 2).

### Synthetic Mucins Enhance *L. fermentum* Adhesion to Mucosa

As lactobacilli are typically cleared by the body in three to 10 days, improving the retention of probiotics in the gut should maximize their beneficial effects.^4,37^ Thus, emerging prebiotics that enhance the adhesion of probiotic lactobacilli to native mucins are under investigation.^68^ To test whether the synthetic mucins could promote adhesion, *L. fermentum* was incubated on a MUC2-modified plate surface to generate a pseudo-mucosa.^69^ AF405 fluorescence confirmed that the synthetic mucin remained post-washing (Figure 5C, Figure S19). Supplementation with synthetic mucin led to enhanced adhesion. The retentive activity of the synthetic mucins was glycan-specific, with enhanced adhesion when polymers with either β-Lac or β-Gal substituents were added (Figure 5B, Figure S19). In contrast, the non-binding polymer with β-GlcNAc residues failed to increase *L. fermentum* adhesion.

### Nutrition of *L. fermentum* via Monovalent and Multivalent Glycans

We next tested whether the synthetic mucin could serve as a nutrient source to cultivate probiotic microbes within the mucosa. Therefore, we assessed microbial growth on the synthetic mucins. *L. fermentum* increased proliferation in the presence of free β-lactose or β-galactose, the same epitopes that increased bacterial binding and adhesion to MUC2 when appended to synthetic mucins (Figure 5D, Figure S20). This trend translated to multivalent scaffolds: MUC2 and the synthetic mucins displaying β-Gal or β-Lac significantly enhanced bacterial growth.

Glycan cleavage could abrogate microbe binding. To assess this possibility, we examined the synthetic mucin composition before and after incubation with cells. A decrease in the glycan substitution was detected, yet some glycans remained (Table S2). These findings suggest that the glycans can be cleaved from the backbone, but that the synthetic mucin will retain its ability to bind microbes.

### Glycosidase Expression and Manipulation

Upon binding to endogenous mucins, bacteria upregulate the production of glycosidases to harvest glycans. We assessed the presence of genes encoding glycosidases of the three lactobacilli. The genomes of *L. reuteri*, *L. fermentum*, and *L. plantarum* were analyzed using the Kyoto Encyclopedia of Genes and Genomes database^70^ to determine the presence of genes encoding glycosidases of interest. All three species encode at least one gene predicted β-galactosidase. To determine whether these glycosidases were expressed and possess the expected enzymatic activity, we used monosaccharides conjugated to the reporter 4-methylumbelliferone (4MU). These substrates were incubated with each bacterial species, and the release of fluorescent 4MU was monitored. When bacteria were grown in nutrient-poor media supplemented with Gal-4MU, they retained efficient galactosidase activity (Figure 5E and Figure S21). No glycosidase activity for α-Man, α-Fuc, or β-GlcNAc was detected with *L. reuteri* or *L. fermentum*. We did observe efficient β-GlcNAc-ase activity with *L. plantarum*, though no binding to the GlcNAc-substituted synthetic mucin was detected (Figure S21).

Because nutrient-rich growth conditions gave rise to nutrient-specific fitness, we investigated whether there were changes in galactosidase expression on a transcriptional level based on growth conditions. We grew *L. fermentum* in a nutrient-rich medium that was supplemented with native or synthetic mucin. Quantitative real-time PCR (qPCR) was used to quantify the relative change in β-galactosidase expression. We found the expression of β-galactosidase genes by *L. fermentum* was increased in either MUC2- or synthetic mucin-doped media (Figure 5F, Figures S22 and S23). Native and synthetic mucins bearing the preferred glycan, β-Gal or β-Lac, enhanced the bacteria’s ability to procure nutrients by increasing its galactosidase expression.

## Discussion

Key features of native mucins are their massive size and the dynamic changes they can undergo. Both contribute to their biological efficacy. Though the molecular mass of synthetic mucins is smaller than that of MUC2, the persistence lengths of native mucin and these analogs are similar, roughly 10 nm.^43,71^ Our observations reveal that the size and complexity of MUC2 is not necessary to bind and cultivate probiotic lactobacilli.

Native mucins cannot be incisively manipulated to interrogate structure–function relationships. Using polymers as mucin analogs provided a synthetically tractable route to glycan-defined scaffolds that could be tuned for bacterial response. Additionally, the polymer synthesis is easily scalable. Though the experiments described use laboratory-scale quantities, the polymerization and subsequent glycosylation of *cis*-poly­(norbornene) could be accomplished on a much larger scale. We limited this initial study to neutral saccharide substituents, but this platform provides the means to add more complex epitopes, including sulfated and sialylated glycans.

In testing the mucin mimics, we found that the polymer backbone was not toxic towards the probiotic lactobacilli, as shown by its enhanced growth in the presence of the synthetic mucin. Further evidence has been published that the poly­(norbornene) scaffold is biocompatible with mammalian cells.^72^ For example, the polymers were not cytotoxic to human vascular endothelial and lung epithelial cells.^73,74^ These data indicate that synthetic polymers can serve as nutrient sources without cytotoxicity.

Bacteria in the mucosal layer typically engage mucins via glycan-binding enzymes and proteins.^75^ We first examined *L. plantarum* as a model probiotic due to its known mannose- and mucin-binding proteins.^49,50,59–62^ In assessing the activity of chemically defined synthetic mucins, we found that *L. plantarum* preferentially binds to α-Man and α-Fuc polymers, regardless of growth phase. These observations are consistent with the similar arrangements of the hydroxyl groups within these two glycan epitopes. Across all studies, α-ArMan polymers outperformed α-PEG_3_Man polymers. Initially designed for binding to dendritic cell lectin DC-SIGN, the arylated α-Man ligand interacts with the mannose residues and aromatic amino acid residues in the binding pocket, which may be occurring in the described mucin- or mannose-binding proteins expressed by bacteria.^76^ Aromatic substituents can enhance the glycan affinity for many lectins.^77–79^ However, to ensure that our synthetic mucins occupied similar binding sites to native mucins, we generally used oligoethylene glycol-linked *O*-glycans.

While it is known that growth medium and strain diversity can influence *L. reuteri* affinity for native mucins,^80,81^ we reasoned that the mucin mimics could reveal differences in glycan specificity. Regardless of the growth phase, *L. reuteri* grown in nutrient-rich conditions preferentially bound polymers bearing α-Fuc or β-Lac, common native mucin termini. In contrast, when the bacteria were grown in nutrient-poor minimal media conditions, probiotic bacteria bound synthetic mucins indiscriminately. Taken together, these data indicate mucin binding of *L. reuteri* depends on bacterial growth conditions and favors promiscuous attachment in nutrient-poor physiological settings, likely contributing to the overall fitness of this probiotic.

Synthetic mucin binding to *L. fermentum* depended less on the glycan epitopes displayed than did *L. reuteri* or *L. plantarum*, the latter bound preferentially to two distinct glycan epitopes. The isolated surface mucin-binding domain MBD_93_ of *L. fermentum* interacted with mucin glycans GalNAc, GlcNAc, and Gal.^66^ These findings align well with the binding profile we observed when *L. fermentum* was grown to mid-log phase. However, the glycan epitope with the highest binding observed for *L. fermentum* was the β-Lac polymer in mid-log phase (97%), outperforming native mucin binding. As future work further characterizes binding profiles of probiotic bacteria, it will be interesting to understand how more complex glycans, such as disaccharides or core glycan sequences, impact binding.

Native mucins can function as prebiotics by increasing the adhesion of probiotic bacteria to the mucosa.^34–36^ We hypothesized that the bacteria would better adhere to mucosa upon addition of synthetic mucin via two different mechanisms: 1) the polymer-mediated clustering of bacteria leads to bacterial entrapment and 2) the probiotic binds to the synthetic and native mucins, generating more anchoring points to the mucosa. Upon generation of a pseudo-mucosa through a mucin-coated plate, we observed that synthetic mucins increased the adhesion of *L. fermentum*. The increased retention of the probiotic to the pseudo-mucosa was glycan-dependent. No polymer fluorescence was detected when synthetic mucins were added in the absence of cells, corroborating our observations that β-Lac and β-Gal polymers are effective binders of *L. fermentum*. These findings suggest that synthetic mucins can act as prebiotics and promote adhesion through glycan-specific binding to facilitate the retention of lactobacilli.

*In vivo*, a microbe’s mucus-binding capacity determines its ability to colonize the mucus and thereby support its growth.^29–32^ Synthetic mucins increase the adhesion and capture of probiotic lactobacilli in a pseudo-mucosa. However, *in vitro* experiments cannot replicate the dynamics of mucus *in vivo*. Some studies indicate that bacterial binding to the mucus can promote clearance. Thus, it remains unclear how mucus binding influences probiotic populations. We envision the synthetic mucins could act as probes to better understand how mucus-binding influences microbial retention.

In addition to improving bacterial adhesion, prebiotics are typically metabolized by probiotic bacteria to encourage growth. To evaluate if the polymers were nutrient sources for probiotic lactobacilli, we compared bacterial growth rates when grown in media supplemented with different glycan additives in the form of a free saccharide or the multivalent synthetic mucin substrates. The growth of *L. fermentum* depended on the identity of the glycan displayed by the mucin mimic, with more cellular growth observed when grown in the presence of β-Gal or β-Lac-bearing compounds. These polymers also effectively interacted with *L. fermentum*, suggesting that mucin mimics displaying these glycans could improve adhesion and function as a nutrient source, thus serving as novel prebiotics.

To access nutrients in the gut, bacteria produce glycosidases to degrade complex glycans to yield sustainable nutrients. Mucin-processing enzymes have been well-characterized for commensals, such as *Akkermansia muciniphila*.^33,82–84^ In contrast, few reports detail the expression of different glycosidases and their dependence on mucins for lactobacilli.^85,86^ Using fluorogenic glycan substrates, glycosidase activity was monitored for *L. plantarum*, *L. reuteri*, and *L. fermentum*. The observed galactosidase activity suggests that *L. reuteri* and *L. fermentum* could use β-Gal or β-Lac synthetic mucins as prebiotics to bind mucin-binding proteins as an anchoring point and cleave these *O*-glycans for nutrients through glycosidases. In addition to galactosidase activity, *N*-acetylglucosaminidase activity was observed for *L. plantarum*. This observation could be relevant to prior reports that show *L. plantarum* encodes a protein with a chitin-binding domain, providing a mechanism to bind poly­(β-GlcNAc).^87^ The glycosidase expression could be manipulated through growth conditions, with increased expression observed on a transcriptional level when native or synthetic mucins were added to the bacterial growth conditions. These data provide further support that the synthetic mucins can cultivate and manipulate probiotic lactobacilli.

## Conclusion

Synthetic mucins are powerful, chemically tractable tools to characterize mucin–probiotic interactions. The glycan specificity was determined for three probiotic lactobacilli using chemically defined mucin surrogates. Mucin mimics bearing relevant binding agents facilitated enhanced adherence, retention, and growth of lactobacilli to mixtures of natural and synthetic mucins, suggesting synthetic mucins can serve as prebiotics. These findings underscore the value of elucidating mucin–bacteria interactions. In this way, mucin surrogates can facilitate the development of supplements that support a healthy microbiome.

## Supplementary Material



## Notes

Experimental procedures, materials, instrumentation, and additional tables and figures including minimal media conditions, bacterial strains, synthetic routes to polymers, flow cytometry data, microscopy and clustering data, adhesion results, OD600 data, glycosidase activity assays, qPCR data, and ^1^H NMR spectra (PDF)

## Notes

Protocols involving samples from human participants were approved by the Massachusetts Institute of Technology’s Committee on the Use of Humans as Experimental Subjects.

## Abbreviations

4-MU: 4-methylumbelliferyl
AF405: AlexaFluor 405
α-ArMan: aryl α-mannosyl
cFDA: carboxyfluorescein diacetate
DMSO: dimethyl sulfoxide
α-Fuc: fucosylated
β-Gal: β-galactosyl
β-GalNAc: *β-N*-acetyl galactosaminyl
β-GlcNAc: β-*N*-acetyl glucosaminyl
HEPES: 4-(2-hydroxyethyl)-1-piperazineethanesulfonic acid buffer
β-Lac: β-lactosyl
MRS: Man, Rogosa, and Sharpe broth
OD600: optical density at 600 nm
PEG_3_: triethylene glycol
qPCR: quantitative polymerase chain reaction
